# Evaluating the Relationship Between Early Non‐Motor Symptoms and Late Diagnosis of Parkinson's Disease Based on the PPMI Database From 2012 to 2018: A Retrospective Cross‐Sectional Study

**DOI:** 10.1002/hsr2.73081

**Published:** 2026-08-26

**Authors:** Shakawat Hossain, Farjana Islam

**Affiliations:** ^1^ Department of Biochemistry and Molecular Biology Shahjalal University of Science and Technology Sylhet Bangladesh

**Keywords:** clinical neurology, diagnosis delay, neurodegenerative disorder, non‐motor symptoms, Parkinson's disease, prodromal Parkinson's

## Abstract

**Background and Aims:**

Parkinson's (PD) diagnosis is frequently delayed, particularly when patients present with non‐motor symptoms (NMS). This study evaluates the association between NMS, socio‐demographic factors, and diagnostic timing in PD using PPMI data. We aimed to determine how clinical and demographic profiles influence the interval between NMS onset and clinical diagnosis, acknowledging that this interval reflects both biological disease progression and healthcare‐related factors.

**Methods:**

A cross‐sectional analysis was conducted on de‐identified data from 1024 PD patients in the PPMI cohort (2012–2018). Multivariable logistic regression was utilized to identify independent factors associated with diagnostic timing (early vs. late). Predictive performance was assessed using the Area Under the ROC Curve (AUC) and calibration analysis. Statistical significance was defined as *p* < 0.05, and an Odds Ratio (OR) > 1 was considered indicative of later diagnostic timing.

**Results:**

Among the 1024 examined patients, multivariable logistic regression indicated that individual early NMS were not independently associated with diagnostic timing (*p* > 0.05 for all). In contrast, specific socio‐demographic factors exhibited significant independent associations: male gender was associated with higher odds of later diagnosis (aOR = 1.48, *p* = 0.0114), while primary education (aOR = 0.29, *p* < 0.001) and unemployment (aOR = 0.53, *p* = 0.0025) were significantly associated with earlier diagnosis. The overall model showed fair‐to‐good discriminative ability with an AUC of 0.692, and no issues with multicollinearity (GVIF ≤ 1.15) were detected.

**Conclusion:**

Socio‐demographic variables, rather than individual non‐motor symptoms, demonstrate the strongest independent associations with diagnostic timing in this cohort. The results suggest that diagnostic delays may be influenced more by gender‐based healthcare‐seeking behaviors and socioeconomic factors than by specific clinical NMS profiles. These findings highlight the need for socio‐demographically tailored screening strategies in the prodromal phase of PD.

## 1 Introduction

Parkinson's disease (PD) is a chronic progressive neurodegenerative disorder that is characterized by dysregulation across the neurotransmitter pathways in the central and autonomic nervous system [1]. While traditionally defined by motor hallmarks such as bradykinesia, rigidity, and resting tremor, PD encompasses a broad spectrum of NMS—including cognitive impairment, autonomic dysfunction, sleep disturbances, fatigue, and mood disorders—that severely impact health‐related quality of life [1]. It has been estimated that the prevalence of PD is 1–2 per 1000 individuals, with an incidence rate of 10–20 per 100,000 people per year [2]. Loss of muscle atonia and abnormal behavior during sleep, identified as rapid eye movement (REM) sleep behavior disorder, is associated with synucleinopathies such as PD, dementia with Lewy bodies, and multiple system atrophy [3]. REM behavior disorder often precedes the clinical diagnosis of PD and is evaluated as a prodromal marker of PD [4, 5]. Furthermore, neuropsychiatric features such as mood disorders exhibit a strong association with structural and functional defects in the prefrontal cortex, hippocampus, or hypothalamus, being heavily linked with clinical depression [6]. The prodromal phase of PD begins several years before the manifestation of motor symptoms. Historically, these symptoms were observed 10–15 years before the onset of motor symptoms. However, clinical studies have predominantly focused on motor manifestations [7, 8]. Overlooking these prodromal symptoms allows neurodegeneration to progress in the brain. Previous research suggests that these NMS are not merely secondary effects of PD; rather, they are integral to the pathogenesis of the disease [9, 10, 11]. In certain instances, NMS poses greater treatment challenges than motor symptoms [12, 13]. The brain regions affected by PD sustain damage before motor symptoms become apparent, which is attributed to the presence of early non‐motor symptoms. Both patients and healthcare providers often overlook these non‐motor symptoms, consequently complicating their diagnosis. By identifying the types and timelines of these non‐motor symptoms, biomarkers, and primary screening tools can be developed. Recognizing the prodromal stage can enhance disease management and potentially delay the progression of PD [14].

Previous research has mainly focused on the motor symptoms of PD. However, a significant gap exists in the literature regarding the progression of NMS and the factors associated with its exacerbation. Although numerous studies have acknowledged the association between NMS and PD, the differences in diagnostic timing serve as a critical factor for the recognition of these symptoms and clinical evaluation. Notably, this diagnostic interval is influenced by a complex interplay of biological disease progression, specifically the time required to reach the “motor threshold” and healthcare‐seeking behavior [15]. Previous literature suggests that socio‐demographic factors, such as age, gender, and socioeconomic status, may further modulate this diagnostic trajectory [16, 17]. However, most clinical studies have focused on the severity of motor symptoms, leaving a significant gap in our understanding of how specific NMS profiles and demographic variables are associated with diagnostic delays [18].

The primary aim of this study is to explicitly evaluate the association between selected NMS and diagnostic timing in PD. We hypothesized that specific prodromal NMS profiles and demographic variables significantly modulate the diagnostic trajectory. Importantly, this study distinguishes diagnostic timing from disease progression and acknowledges that the observed interval may reflect both biological progression (specifically the time required for the emergence of motor symptoms necessary for clinical diagnosis) and healthcare‐related factors.

## 2 Result

### 2.1 Demographic and Clinical Characteristics of the Participants

A total of 1024 participants were analyzed, with a mean age of 65.9 ± 10.8 years (*p *= 0.440 between groups). The cohort consisted of 520 males (50.8%) and 504 females (49.2%). Significant gender differences were observed between early and late diagnosis groups (*p *= 0.045), as detailed in Table 1. The overall median diagnosis delay was 12.5 months (IQR: 7.0–26.0). The early diagnosis group had a significantly shorter median delay of 7.0 months (IQR: 4.0–10.0) compared to 26.0 months (IQR: 17.0–37.0) in the late diagnosis group (*p* < 0.001).

**Table 1:** The demographic and clinical characteristics of the participants are presented in this table, detailing age by sex and the distribution of residence, educational levels, and occupation for both male and female participants.

Patient characteristics	Overall (*N* = 1024)^a^	Early *N* = 512^a^	Late *N* = 512^a^	*p* value^b^
Age (years)	65.9 ± 10.8	65.7 ± 11.3	66.2 ± 10.3	0.440
Gender				0.045
Female	504.0 (49.2%)	268.0 (52.3%)	236.0 (46.1%)	
Male	520.0 (50.8%)	244.0 (47.7%)	276.0 (53.9%)	
Residence area				0.011
Rural	305.0 (29.8%)	134.0 (26.2%)	171.0 (33.4%)	
Urban	719.0 (70.2%)	378.0 (73.8%)	341.0 (66.6%)	
Education level				< 0.001
Graduate	328.0 (32.0%)	137.0 (26.8%)	191.0 (37.3%)	
No school	71.0 (6.9%)	27.0 (5.3%)	44.0 (8.6%)	
Postgraduate	97.0 (9.5%)	35.0 (6.8%)	62.0 (12.1%)	
Primary	294.0 (28.7%)	220.0 (43.0%)	74.0 (14.5%)	
Secondary	234.0 (22.9%)	93.0 (18.2%)	141.0 (27.5%)	
Occupation type				< 0.001
Desk job	197.0 (19.2%)	79.0 (15.4%)	118.0 (23.0%)	
Manual	242.0 (23.6%)	87.0 (17.0%)	155.0 (30.3%)	
Retired	289.0 (28.2%)	158.0 (30.9%)	131.0 (25.6%)	
Unemployed	296.0 (28.9%)	188.0 (36.7%)	108.0 (21.1%)	
Diagnosis delay (months)	12.5 (7.0, 26.0)	7.0 (4.0, 10.0)	26.0 (17.0, 37.0)	< 0.001

^a^ Mean ± SD; *n* (%); median (Q1, Q3).

^b^ Welch two‐sample *t*‐test; Pearson's *χ* ^2^ test; Wilcoxon rank sum test.

### 2.2 Associated Factors of Diagnostic Timing: Logistic Regression Analysis

The multivariable logistic regression model, adjusted for all potential confounders, including age, sex, education level, occupation, and residence, indicated that individual early NMS were not independently associated with diagnostic timing (*p *> 0.05 for all variables). Specifically, the adjusted odds ratio (aOR) for the eight examined symptoms were as follows: smell loss (aOR = 1.02, 95% CI 0.75–1.38, *p* = 0.8997), constipation (aOR = 1.07, 95% CI 0.80–1.42, *p* = 0.6592), REM sleep disorder (aOR = 1.36, 95% CI 0.98–1.89, *p* = 0.0627), mood disorder (aOR = 0.85, 95% CI 0.63–1.14, *p* = 0.2756), fatigue (aOR = 1.06, 95% CI 0.79–1.43, *p* = 0.6942), cognitive impairment (aOR = 1.07, 95% CI 0.78–1.45, *p* = 0.6868), urinary dysfunction (aOR = 1.21, 95% CI 0.86–1.71, *p* = 0.2673), and sexual dysfunction (aOR = 0.81, 95% CI 0.60–1.11, *p* = 0.1872) (Table 2).

**Table 2:** Predictors of delayed diagnosis from multivariable logistic regression analysis.

Non‐motor symptom/variable	aOR^a^	95% CI	*p* value	GVIF^b^
Early non‐motor symptoms				
Smell loss	1.02	0.75–1.38	0.8997	1.14
Constipation	1.07	0.80–1.42	0.6592	1.07
REM sleep disorder	1.36	0.98–1.89	0.0627	1.12
Mood disorder	0.85	0.63–1.14	0.2756	1.13
Fatigue	1.06	0.79–1.43	0.6942	1.13
Cognitive impairment	1.07	0.78–1.45	0.6868	1.11
Urinary dysfunction	1.21	0.86–1.71	0.2673	1.07
Sexual dysfunction	0.81	0.60–1.11	0.1872	1.15
Socio‐demographics				
Age	1.00	0.99–1.01	0.5440	1.09
Gender (male)^c^	1.48	1.09–1.99	0.0114*	1.14
Education (primary)	0.29	0.20–0.42	< 0.001***	1.08
Occupation (unemployed)	0.53	0.36–0.80	0.0025**	1.05
Residence (urban)	0.76	0.56–1.03	0.0726	1.04

^a^ Model adjustment: The multivariable model was simultaneously adjusted for all listed non‐motor symptoms and socio‐demographic covariates (age, gender, education level, occupation type, and residence).

^b^ Multicollinearity: Multicollinearity was assessed using Generalized Variance Inflation Factors (GVIF). All values were ≤ 1.15, indicating no significant collinearity issues.

^c^ Statistical interpretation: Given the exploratory nature of this analysis and the use of multiple testing, results were interpreted cautiously to minimize false‐positive risks.

* *p* < 0.05

** *p* < 0.001

*** *p* < 0.01.

In contrast, specific socio‐demographic factors emerged as being significantly and independently associated with diagnostic timing. Being male was associated with significantly higher odds of later diagnosis (aOR = 1.48, 95% CI 1.09–1.99, *p* = 0.0114). Conversely, individuals with a primary education (aOR = 0.29, 95% CI 0.20–0.42, *p* < 0.001) and those who were unemployed (aOR = 0.53, 95% CI 0.36–0.80, *p* = 0.0025) showed significantly lower odds of delayed diagnosis. All Generalised Variance Inflation Factor (GVIF) values were ≤ 1.15, confirming the absence of multicollinearity issues within the model.

To evaluate the statistical robustness of the pragmatic 12‐month binary threshold, a sensitivity analysis was conducted using a multivariable linear regression model with diagnostic delay treated as a continuous variable (in months) (Supporting Information S1: Table S1). The continuous model strongly cross‐validated the primary socio‐demographic findings from the logistic regression. Specifically, primary education remained highly and independently associated with significantly reduced diagnostic timing compared to the graduate reference group (*β* = −8.1, 95% CI: −10 to −5.9, *p* < 0.001). Interestingly, when evaluated across the full continuous spectrum, smell loss emerged as a statistically significant clinical factor that independently increased the diagnostic delay by approximately 1.9 months (*β* = 1.9, 95% CI: 0.16–3.6, *p* = 0.032). Conversely, the independent association with male gender observed in the dichotomized logistic model became non‐significant in the continuous framework (*β* = 0.16, *p* = 0.854), indicating that while gender splits patients across the 1‐year threshold, the overall continuous variance in diagnostic delay is more heavily driven by educational level and specific prodromal olfactory deficits.

### 2.3 Exploratory Visualization of NMS Associated With Diagnostic Timing

**Figure 1:**
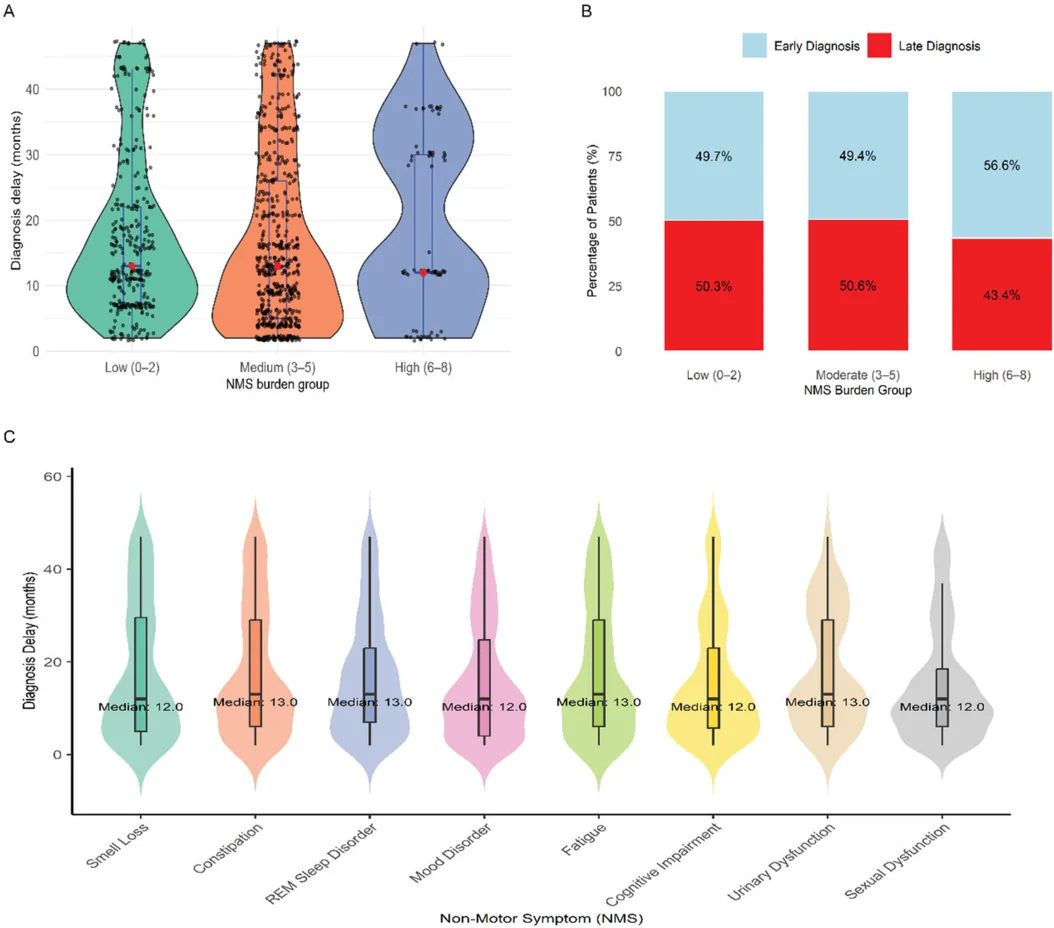
Distribution of diagnostic timing and delay patterns across Non‐Motor Symptom (NMS) profiles. (A) Violin plot showcasing the distribution of diagnosis delay (in months) stratified by cumulative NMS burden groups: low (0–2 symptoms), medium (3–5 symptoms), and high (6–8 symptoms). Jittered dots represent individual patient records (*n* = 1024), internal blue boxplots indicate the interquartile ranges (IQR), and solid red circles mark the median delay values. (B) Stacked bar plot illustrating the proportion (%) of early (≤ 12 months; light blue) versus late (> 12 months; red) diagnostic timing within each NMS burden strata. Proportional variations (e.g., 50.3% in low, 50.6% in moderate, and 43.4% in high burden) were statistically evaluated using the *χ* ^2^ test. (C) Symptom‐specific violin plots displaying the right‐skewed distribution of diagnostic delay for each of the eight individual non‐motor symptoms. Solid horizontal indicators within each profile explicitly denote the median values (ranging between 12.0 and 13.0 months consistently). Group comparisons across continuous metrics were performed via the non‐parametric Mann–Whitney U test following normality verification using the Shapiro–Wilk test.

To understand the distribution of diagnosis timing across different NMS burden groups, we generated violin plots using non‐parametric methods for group comparisons (Figure 1A). As the Shapiro–Wilk test indicated that the diagnostic timing variable was not normally distributed (*p* < 0.05), we reported the median and interquartile range (IQR) for each group. The median diagnostic timing remained relatively consistent across burden levels, with the low‐burden group (0–2 symptoms) showing a median of 13.0 months and the high‐burden group (6–8 symptoms) showing a median of 12.0 months. These findings suggest that while a higher cumulative NMS burden is a characteristic of the cohort, it does not lead to a linear increase in the median delay.

We then compared the early and late diagnostic segments according to NMS load categories (Figure 1B). Patients with the highest symptom burden (high: 6–8 symptoms) had a lower percentage of later diagnostic timing at 43.4% (*n* = 106/244), whereas those with low and moderate NMS burdens had higher percentages at 50.3% (*n* = 144/286) and 50.6% (*n* = 250/494), respectively. Despite a large cumulative burden, this suggests that more numerous manifestations may prompt earlier therapeutic attention. To further assess individual symptoms, violin plots showed that the median delay for each of the eight NMS consistently ranged between 12.0 and 13.0 months (Figure 1C). Overall, the distribution of diagnostic timing was right‐skewed, with most patients receiving a diagnosis within the 12 to 24‐month interval.

### 2.4 Multivariable Regression Analysis of Diagnostic Timing

**Figure 2:**
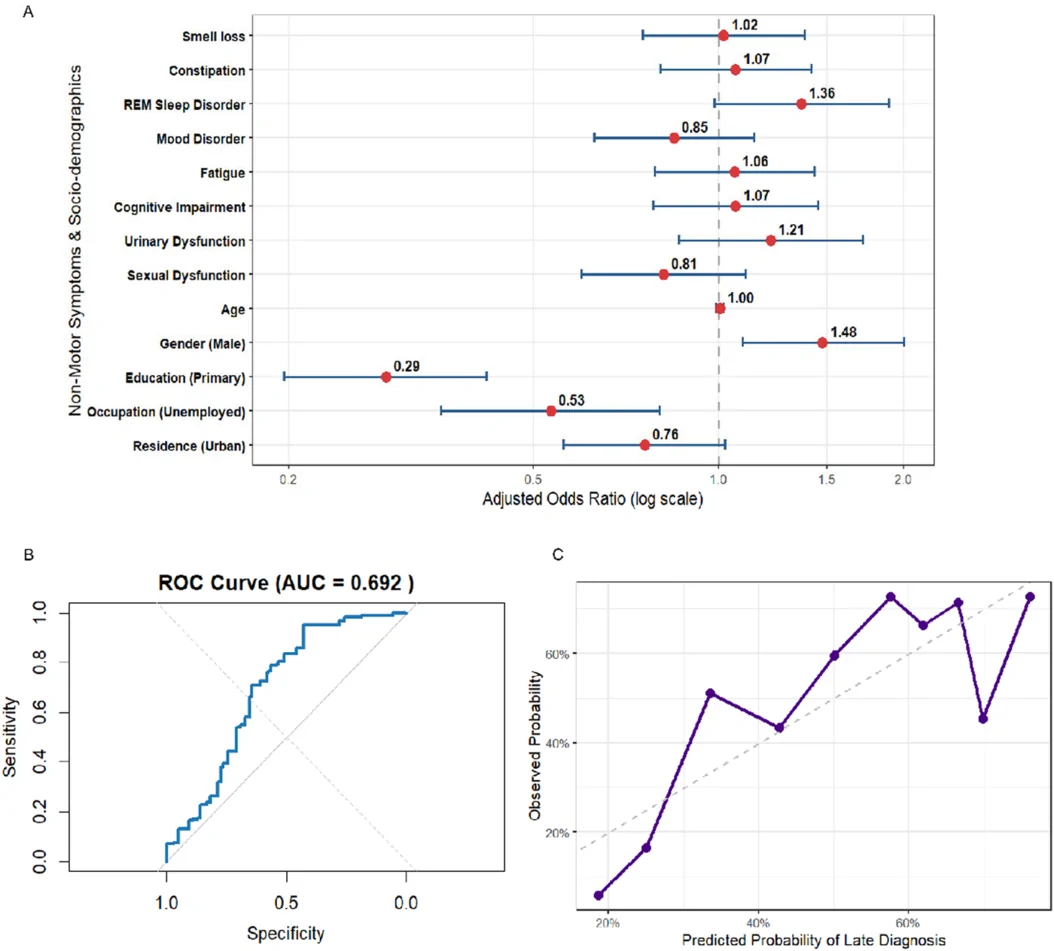
Multivariable logistic regression model outcomes and predictive performance diagnostics. (A) Forest plot illustrating the adjusted odds ratios (aOR) and 95% confidence intervals (CIs) on a log scale for both the eight primary non‐motor symptoms and the fully adjusted socio‐demographic covariates, systematic with the data ordering in Table 2. An aOR > 1 denotes higher odds of late diagnosis (> 12 months). (B) Receiver operating characteristic (ROC) curve evaluating the model's overall discriminative capacity, demonstrating a moderate predictive performance with an area under the curve (AUC) of 0.692. (C) Calibration curve plotting the predicted versus observed probabilities of late diagnosis across the patient cohort, demonstrating robust model alignment and baseline reliability, particularly within the mid‐range probability zones.

The multivariable logistic regression model was used to evaluate the independent associations of early NMS and socio‐demographic factors on diagnostic timing. The forest plot of the aOR for the NMS confirmed that none of the individual clinical features was independently associated with diagnostic timing when fully adjusted for covariates (*p *> 0.05), although REM sleep disorder (aOR = 1.36) and urinary dysfunction (aOR = 1.21) showed the highest relative odds for later diagnosis within the symptom cluster (Figure 2A).

The overall predictive performance of the multivariable model, as assessed by the Area Under the ROC Curve (AUC), was 0.692, indicating a moderate discriminative ability in distinguishing between early and late diagnostic groups (Figure 2B). This cross‐validated performance suggests that while the model captures meaningful clinical and demographic trend lines, it should be interpreted cautiously as an exploratory profiling tool rather than a definitive diagnostic classifier. Furthermore, calibration analysis demonstrated that the predicted probabilities of later diagnosis were well‐aligned with observed outcomes, particularly within the mid‐range probability zones, suggesting that the model is reliable for identifying clinical trends despite some variability at the higher probability thresholds (Figure 2C).

### 2.5 Symptom‐Specific Association Strength and Significance

**Figure 3:**
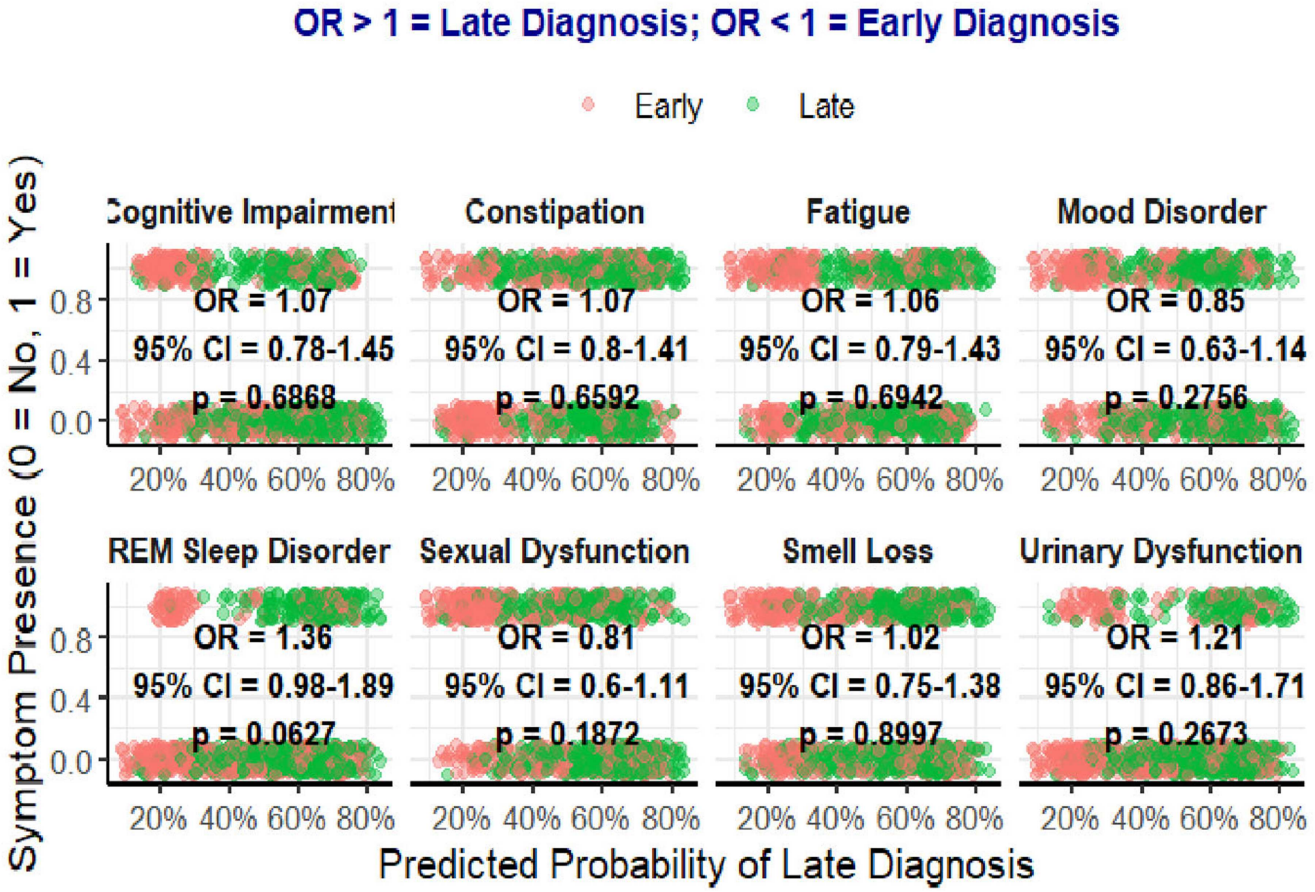
Distribution of predicted probabilities and statistical significance for individual non‐motor symptoms (NMS). Jittered dot plots illustrate the predicted probability of late diagnosis across eight individual non‐motor symptoms: cognitive impairment, constipation, fatigue, mood disorder, REM sleep disorder, sexual dysfunction, smell loss, and urinary dysfunction. The x‐axis represents the predicted probability of late diagnosis (ranging from 20% to 80%), and the y‐axis indicates symptom presence (0 = no, 1 = yes). Individual data points are color‐coded to differentiate patient groups, where red dots represent early diagnosis and green dots represent late diagnosis. Each panel explicitly displays the adjusted odds ratio (OR), 95% confidence interval (CI), and associated *p* value calculated via multivariable logistic regression after adjusting for socio‐demographic confounders. An OR > 1 indicates an increased likelihood of late diagnosis, whereas an OR < 1 indicates a trend toward early diagnosis. As shown, none of the individual symptoms reached independent statistical significance (*p* > 0.05). Abbreviations: CI, confidence interval; NMS, non‐motor symptom; OR, odds ratio; REM, rapid eye movement.

To evaluate the relationship between individual NMS and the probability of a later PD diagnosis, we analyzed symptom‐specific association strengths using jittered probability plots (Figure 3). Based on the fully adjusted multivariable logistic regression analysis, none of the eight examined symptoms demonstrated a statistically significant independent association with diagnostic timing (*p* > 0.05 for all). However, visual assessment of the distribution trends indicated that REM sleep disorder (OR = 1.36, 95% CI: 0.98–1.89, *p* = 0.0627) and urinary dysfunction (OR = 1.21, 95% CI: 0.86–1.71, *p* = 0.2673) trended toward a higher likelihood of later diagnosis, while sexual dysfunction (OR = 0.81, *p* = 0.1872) and mood disorder (OR = 0.85, *p* = 0.2756) showed trends toward earlier diagnosis. These findings reinforce that when social and demographic factors are accounted for, individual clinical symptoms alone do not exhibit robust independent associations with diagnostic timing in this cohort.

## 3 Discussion

In this retrospective study, we evaluated the association between early NMS, socio‐demographic factors, and diagnostic timing in PD. Our multivariable analysis revealed that while individual NMS—such as constipation and REM sleep behavior disorder were associated with a higher likelihood of being in the later diagnosis group, they did not retain independent associations when adjusted for confounders. Instead, socio‐demographic factors, specifically male gender, education level, and employment status, exhibited the strongest independent associations with diagnostic timing in this cohort.

The lack of a statistically significant independent association between specific NMS and diagnostic timing suggests that the clinical journey to PD is multifaceted. Our findings regarding NMS are largely consistent with previous literature, suggesting that while symptoms such as constipation and REM sleep behavior disorder are well‐established prodromal markers of synucleinopathy [19, 20], their non‐specific nature in primary care settings often leads to them being attributed to general age‐related or systemic issues rather than neurological pathology [21]. While our cross‐sectional data show a significant association with diagnostic timing, these patterns should be interpreted as concurrent markers rather than a confirmed temporal sequence. This often results in a “clinical overshadowing” where the significance of these symptoms is only recognized retrospectively after the emergence of motor signs.

Conversely, our multivariable analysis highlighted that male gender, higher education levels, and active employment status were significantly associated with a shorter diagnostic timing interval. Several potential reasons could explain these socio‐demographic trajectories. First, regarding sex‐based differences, males with PD frequently present with more prominent or rapidly progressive motor symptoms early on, which might breach the diagnostic “motor threshold” quicker than in females, thereby accelerating formal clinical evaluation [22]. Additionally, gendered variations in healthcare utilization and distinct symptom‐reporting behaviors may prompt earlier consultations [23]. Second, higher educational attainment is often tightly linked with greater health literacy; highly educated individuals may recognize subtle, non‐motor cognitive or autonomic shifts as pathological rather than normal ageing, prompting them to seek specialist care sooner [24]. Finally, active employment status serves as a critical functional baseline. Individuals engaged in regular professional occupations are highly sensitive to minor declines in cognitive speed, fatigue management, or fine motor skills, as these impairments directly interfere with daily workplace productivity, forcing an earlier medical consultation compared to those who are retired or unemployed [25]. Rather than reflecting intrinsic biological pathways, these socio‐demographic links function as vital proxies for systemic factors, including healthcare accessibility, socioeconomic safety nets, and occupational demands that heavily modulate the clinical diagnostic timeline.

Our multivariable regression model demonstrated a moderate discriminative ability with an AUC of 0.692, which represents an incremental improvement over simpler clinical models. This indicates that while NMS profiles offer valuable insights, they must be interpreted alongside socio‐demographic contexts to accurately map the diagnostic trajectory. However, given that an AUC of 0.692 denotes moderate rather than high discrimination, these predictive thresholds must be interpreted with caution. The calibration of our model further suggests that it is particularly reliable in the mid‐range probability zones, making it a potentially useful tool for identifying general risk trends regarding diagnostic delay rather than delivering precise individual‐level classification.

### 3.1 Limitations

This study has several limitations. Due to its cross‐sectional design, a definitive temporal relationship or causality between symptom onset and diagnostic timing cannot be established. The observed interval likely reflects a combination of biological disease progression (the time required to reach the “motor threshold”) and healthcare‐seeking behavior. Furthermore, the PPMI cohort is derived from specialized research centers and may not be fully representative of the general population. Referral bias may also exist, as specific symptoms—such as mood disorders or sexual dysfunction—may prompt earlier clinical consultation independently of underlying disease biology. Consequently, this study cannot clearly distinguish between true diagnostic delay and the delayed emergence of motor symptoms required for a PD diagnosis. While our model's performance is robust (AUC 0.692), these findings should be considered hypothesis‐generating, and future prospective studies are needed to fully elucidate these complex interplays. Furthermore, because the timeline for early non‐motor symptom onset is entirely patient‐reported, this study is inherently susceptible to recall bias, which may introduce uncertainty into the exact prodromal duration.

## 4 Conclusion

This study identifies significant associations between socio‐demographic factors, specific NMS, and diagnostic timing in PD. However, these findings should be interpreted cautiously, given the cross‐sectional design. Further longitudinal studies are required to validate these associations and better understand the dynamic relationship between symptom onset and clinical diagnosis.

## 5 Methods and Materials

### 5.1 Study Design and Data Source

**Figure 4:**
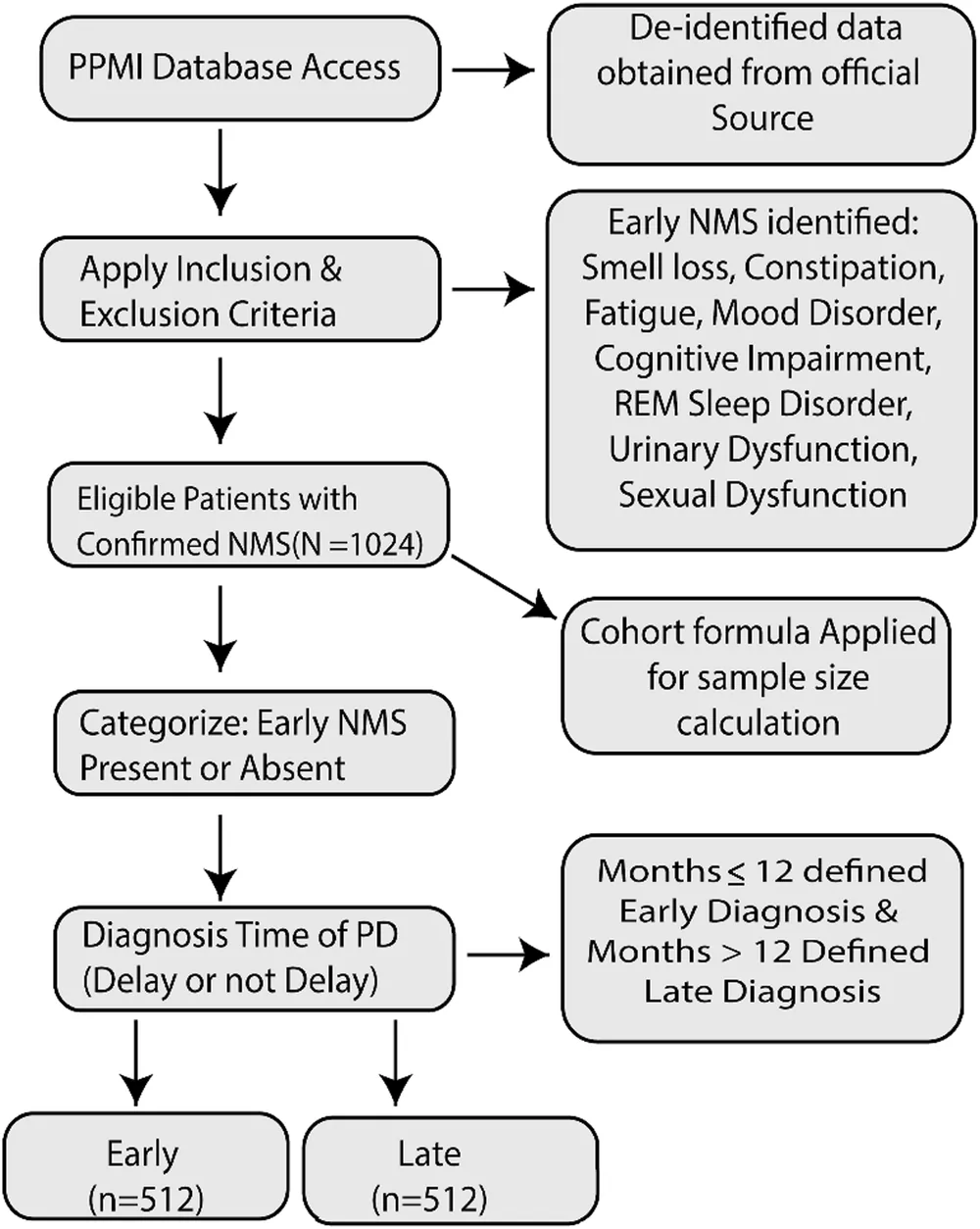
Study flow‐chart and patient categorization by diagnostic timing in Parkinson's disease. A total of 1024 participants with confirmed non‐motor symptoms were identified from the PPMI database. Participants were categorized into two groups based on the interval between symptom onset and clinical PD diagnosis: Early diagnosis (≤ 12 months; *n* = 512) and Late diagnosis (> 12 months; *n* = 512). This classification served as the framework for all subsequent delay‐related analyses.

This retrospective cross‐sectional study used data obtained from the PPMI, a publicly accessible secondary database. All data were de‐identified and made available for research purposes with approval from the data‐sharing committee (Figure 4).

### 5.2 Inclusion and Exclusion Criteria

Participants were selected from the PPMI database based on strict eligibility requirements to ensure data consistency and minimize confounding effects.

### 5.3 Inclusion Criteria

Patients diagnosed with idiopathic PD based on the criteria established by the Michael J. Fox Foundation (USA) from 2012 to 2018. Availability of comprehensive diagnostic records, including historical data on NMS before the formal diagnosis. Individuals aged 40 years and older. Complete documentation of baseline socio‐demographic covariates, including age, sex, education level, type of occupation, and place of residence.

### 5.4 Exclusion Criteria

Individuals with atypical or secondary Parkinsonism, including drug‐induced and vascular Parkinsonism. Pre‐existing neurological or psychiatric disorders (such as schizophrenia and major depressive disorder) that may confound symptom reporting. Individuals were excluded from the study if they presented with atypical or secondary Parkinsonism, including drug‐induced and vascular Parkinsonism. Patients with pre‐existing neurological or psychiatric disorders, such as schizophrenia and major depressive disorder, that could confound symptom reporting were also excluded. Additionally, individuals with genetic or familial forms of PD, as well as those with severe systemic comorbidities—including chronic renal failure, pulmonary disorders, anemia, heart failure, infectious diseases, and gastrointestinal disorders—were not included. Finally, cases involving incomplete medical records or missing data on key clinical variables, such as symptom onset or diagnostic timing, were excluded from the analysis.

### 5.5 Study Population and Sampling

Eligible participants from the Parkinson's Progression Markers Initiative (PPMI) dataset were identified through purposive sampling, yielding a final analytical cohort of 1024 patients who strictly met all selection criteria. Only participants with complete records of both early non‐motor symptoms (NMS) and comprehensive diagnostic data were included. The dataset was organized in a standardized CSV file format.

Cochran's formula was employed to determine the required sample size:

$$n=\frac{Z^{2}.p.\left(1-p\right)}{e^{2}}$$

$$n=\frac{{\left(1.96\right)}^{2}.0.6.\left(1-0.6\right)}{{\left(0.03\right)}^{2}}=1024$$

Where a baseline prevalence (*p*) of 60% was assumed based on the literature [26, 27], a *Z*‐value of 1.96 corresponding to a 95% confidence level, and a margin of error (e) of 0.03 [26]. The precision was targeted with a margin of error of 0.03 due to resource constraints and the exploratory nature of the study.

### 5.6 Data Variables

Demographic variables included age at diagnosis, sex (male/female), educational level (primary, secondary, no formal education, graduate), occupation (desk job, retired, manual labor, unemployed), and residence (rural or urban). Clinical variables encompassed diagnostic timing, defined as the duration (in months) between the patient‐reported retrograde onset of the first non‐motor symptom and the formal clinical diagnosis of PD. A pragmatic 12‐month cutoff was used to categorize diagnostic timing into earlier (≤12 months) versus later (>12 months). This threshold was chosen based on reported onset‐to‐evaluation intervals, which typically cluster around 1 year in real‐world cohorts [28]. Clinically, a 12‐month benchmark serves as a vital indicator in practice to differentiate standard presentation pathways from prolonged trajectories, establishing a critical window for timely therapeutic stratification and early specialist intervention.

NMS were assessed using validated instruments available in the PPMI dataset. Constipation and urinary dysfunction were evaluated using items from the Scales for Outcomes in PD–Autonomic (SCOPA‐AUT) scale [29]. REM sleep behavior disorder was assessed using the REM Sleep Behavior Disorder Screening Questionnaire (RBDSQ) [30]. Olfactory dysfunction was evaluated using the University of Pennsylvania Smell Identification Test (UPSIT) [31]. Mood disorders were assessed using standardized depression scales such as the Geriatric Depression Scale (GDS) [32]. Cognitive impairment was evaluated using the Montreal Cognitive Assessment [33]. Fatigue and sexual dysfunction were identified based on patient‐reported items from the PPMI dataset.

Additionally, an NMS Burden Index was constructed by summing the total number of NMS present in each participant. Each symptom was coded as a binary variable (present/absent), and the resulting cumulative score represented the overall burden of non‐motor manifestations. The NMS burden index was subsequently analyzed as a continuous variable in exploratory analyses to evaluate its association with diagnostic delay patterns. Specifically, the cumulative index score (ranging from 0 to 8) was categorized into three distinct burden groups to reflect clinical progression: low burden (0–2 symptoms), moderate burden (3–5 symptoms), and high burden (6–8 symptoms).

### 5.7 Data Analysis

Statistical analysis and visualization were conducted using the R programming language (version 4.5.1). Categorical variables were expressed as frequencies (*n*) and percentages (%). For continuous variables, the Shapiro–Wilk test was initially performed to assess the normality of the distribution. Normally distributed data (e.g., age) were summarized using mean ± standard deviation (SD), while non‐normally distributed data were presented as median and interquartile range (IQR) to provide a robust measure of uncertainty, as suggested by the editorial guidelines. Group comparisons for categorical data were performed using the *χ* ^2^ test. For continuous variables, the independent *t*‐test was used for normally distributed data, while the Mann–Whitney U test was employed for non‐parametric comparisons.

Regarding missing data, a complete‐case analysis approach was adopted since the missingness across key covariates in our PPMI cohort subset was negligible (< 5%), minimizing potential selection bias. To identify independent factors associated with diagnostic timing (> 12 months vs. ≤ 12 months), we constructed a fully adjusted multivariable logistic regression model. This model simultaneously included all primary NMS and was adjusted for a complete set of socio‐demographic covariates (age, sex, residence, education level, and occupation). Multi‐level categorical variables were entered into the model as factors using standard dummy coding. To ensure epidemiological logic, “No School” was designated as the reference category for education level (contrasting against Primary, Secondary, Graduate, and Postgraduate tiers), while “Unemployed” served as the reference category for occupation type. Prior to modeling, standard logistic regression assumptions were rigorously verified: the linearity of continuous predictors (age) against the logit of the outcome was confirmed using the Box–Tidwell transformation (*p* > 0.05), and multicollinearity was assessed using Generalized Variance Inflation Factors (GVIF), with values ≤ 1.15 indicating no significant collinearity issues.

To evaluate the internal validity of the model and guard against overfitting, a 10‐fold cross‐validation procedure was performed to compute the out‐of‐sample predictive performance. All statistical tests were two‐sided, and a significance level of *p* < 0.05 was considered statistically significant. Given the exploratory nature of these multiple comparisons, findings were interpreted with caution regarding potential false positives. OR > 1 is defined as late diagnosis, and OR < 1 as early diagnosis. Finally, to rigorously test the robustness of this binary threshold and counteract any potential loss of statistical information caused by dichotomization, a sensitivity analysis was executed. This involved constructing a multivariable linear regression model treating the absolute diagnostic timing interval strictly as a continuous dependent variable (in months) against the identical set of clinical and socio‐demographic covariates, with model outputs compiled comprehensively in Supporting Information S1: Table S1.

## Author Contributions

**Shakawat Hossain:** conceptualization, methodology, software, data curation, formal analysis, visualization, validation, writing – original draft. **Farjana Islam:** writing – original draft, writing – review and editing, supervision, data curation, investigation, conceptualization, software.

## Funding

The authors have nothing to report.

## Ethics Statement

This analysis was conducted by the Parkinson's Progression Markers Initiative (PPMI), which is publicly accessible at http://www.ppmi-info.org. The PPMI documentation received approval from Institutional Review Boards and Ethics Committees. All participants provided written informed consent for the PPMI study in accordance with the Declaration of Helsinki (Brazil, 2013). The study involved only secondary analysis of de‐identified PPMI data, which had already been registered and approved by agreement. Consequently, no additional ethical approval was required for this analysis. This study adhered to established policies, and no attempts were made to re‐identify participants. I gratefully acknowledge PPMI for granting access to the dataset for this study. A list of funding partners can be found at http://www.ppmi-info.org/fundingpartners.

## Conflicts of Interest

The authors declare no conflicts of interest.

## Transparency Statement

Farjana Islam affirms that this manuscript is an honest, accurate, and transparent account of the study being reported; that no important aspects of the study have been omitted; and that any discrepancies from the study as planned have been explained.

## Supporting information


Supporting File

## Data Availability

Data used in this study are available upon request from the Parkinson's Progression Markers Initiative (PPMI) website (http://www.ppmi-info.org).
