# Supplementary material for: Evaluating the Relationship Between Early Non‐Motor Symptoms and Late Diagnosis of Parkinson's Disease Based on the PPMI Database From 2012 to 2018: A Retrospective Cross‐Sectional Study
Source: Health Sci Rep. 2026 Aug 26;9(9):e73081. doi: 10.1002/hsr2.73081 (PMC13509015; doi:10.1002/hsr2.73081)
Supplement: Supplementary file 1 [file HSR2-9-e73081-s001.docx]

**Supplementary Table S1:** Sensitivity analysis using multivariable linear regression (Continuous outcome: Diagnosis Delay in Months)

| Characteristic | β | 95% CI | p-value |
| --- | --- | --- | --- |
| Smell Loss | 1.9 | 0.16, 3.6 | 0.032 |
| Constipation | 0.84 | -0.79, 2.5 | 0.312 |
| REM Sleep Disorder | -0.55 | -2.4, 1.3 | 0.562 |
| Mood Disorder | -0.79 | -2.5, 0.95 | 0.373 |
| Fatigue | 1.2 | -0.47, 2.9 | 0.156 |
| Cognitive Impairment | -0.39 | -2.2, 1.4 | 0.666 |
| Urinary Dysfunction | 1.3 | -0.67, 3.2 | 0.199 |
| Sexual Dysfunction | -1.2 | -2.9, 0.60 | 0.195 |
| Age | 0.00 | -0.07, 0.08 | 0.913 |
| Gender |  |  |  |
| Female | — | — |  |
| Male | 0.16 | -1.6, 1.9 | 0.854 |
| Education Level |  |  |  |
| Graduate | — | — |  |
| No School | 2.7 | -0.68, 6.1 | 0.118 |
| Postgraduate | 0.80 | -2.4, 4.0 | 0.621 |
| Primary | -8.1 | -10, -5.9 | <0.001 |
| Secondary | 0.48 | -1.7, 2.7 | 0.665 |
| Occupation Type |  |  |  |
| Desk Job | — | — |  |
| Manual | 1.3 | -1.1, 3.8 | 0.292 |
| Retired | -0.51 | -2.8, 1.8 | 0.667 |
| Unemployed | -0.77 | -3.2, 1.6 | 0.530 |
| Residence |  |  |  |
| Rural | — | — |  |
| Urban | 1.3 | -0.42, 3.0 | 0.139 |
| Abbreviation: CI = Confidence Interval | | | |
